# Optimization of a New High Rotary Missile-Borne Stabilization Platform

**DOI:** 10.3390/s19194143

**Published:** 2019-09-24

**Authors:** Xiaokai Wei, Jie Li, Debiao Zhang, Kaiqiang Feng, Jiayu Zhang, Jinqiang Li, Zhenglong Lu

**Affiliations:** 1 National Key Laboratory for Electronic Measurement Technology, North University of China, Taiyuan 030051, China; weixiaokai1128@163.com (X.W.); zhangdebiao@aliyun.com (D.Z.); b1506011@st.nuc.edu.cn (K.F.); 18734196406@163.com (J.Z.); Leejq0302@163.com (J.L.); lzl893633776@gmail.com (Z.L.); 2 Key Laboratory of Instrumentation Science & Dynamic Measurement, North University of China, Taiyuan 030051, China

**Keywords:** inertial measurement system, high-rotation ammunition, roll stabilized platform, bearing nested structure, friction moment

## Abstract

The passive semi-strapdown roll stabilized platform is an inertial platform, which can isolate the rolling of a projectile body by a special mechanical device. In the passive semi-strapdown roll stabilized platform, the bearing device plays an important role in isolating the rolling of the projectile body. The smaller the friction moment of bearing, the smaller the swing angular velocity of the platform, the smaller the range of inertial sensors required, the higher the accuracy of the navigation solution. In order to further reduce the swing angular velocity of the platform and improve the navigation accuracy, the bearing nested structure that could reduce the friction torque is proposed. Combined with the working principle of the passive semi-strapdown roll stabilized platform, the mechanical calculation model of friction at the moment of bearing the nested structure was established. A series of simulation analysis and tests showed that the output stability value of the friction moment was 47% that of a single bearing; the roll rate of the platform based on the bearing nested structure decreased to 50% of that based on the single bearing structure; the position and attitude errors measured of the platform based on the bearing nested structure decreased to more than 50% of that based on the single bearing structure. It showed that the bearing nested structure could effectively reduce the friction moment, improve the axial reliability of the bearing, and provide a more stable working environment for the passive semi-strapdown roll stabilized platform.

## 1. Introduction

In the future, high-tech informationized local wars will be transformed from a highly mechanized unit weapon platform to a highly informationalized weapon system network [1,2]. Precision guided ammunition has been widely used and developed in modern warfare for its advantages of high strike precision, good combat effectiveness, and being able to strike moving targets. In order to meet the needs of modern warfare, the guided transformation of conventional ammunition has become an important trend in the development of conventional ammunition [3].

The realization of conventional ammunition guidance depends on the real-time and accurate measurement of projectile flight navigation parameters. Therefore, how to measure projectile flight navigation parameters reliably and accurately is one of the key technologies of conventional ammunition guidance [4]. The inertial navigation system (INS) based on Newton’s second law can realize autonomous, reliable, and covert three-dimensional navigation positioning and attitude determination under all-weather conditions, globally, and in any environment. The INS is an indispensable and irreplaceable navigation instrument in many precision-guided weapons because it has the characteristics of autonomy, concealment, and acquisition of full motion information of the carrier [5,6].

Since the 1950s, the measurement of navigation parameters of guided ammunition has been a frontier issue concerned by many scholars. The launching and flying environment of high-speed rotating ammunition has the characteristics of high rotation, high overload, and high dynamic, which brings serious challenges to the navigation system [7,8,9]. High-speed rotating ammunition has high angular velocity on the roll axis. In this case, the platform inertial navigation system (PINS) and strapdown inertial navigation system (SINS) are difficult to adapt to the high dynamic and high-speed rotating missile-borne environment, and difficult to measure reliably and accurately [10]. The mismatch between range and accuracy requirements makes the strapdown inertial navigation system unable to accurately measure the axial angular velocity and establish an accurate attitude matrix, so the strapdown inertial navigation system cannot be effectively used in the parameter measurement of high-spin ammunition [11,12,13].

At present, the research on conventional rotating ammunition navigation technology mainly includes integrated navigation, geomagnetic navigation, the gyroscope-free strapdown inertial navigation system (GFSINS), and a single-axis motor stabilized navigation platform [14]. With the development of the global navigation satellite system (GNSS) and inertial navigation technology, INS/GNSS integrated navigation technology is continuously applied in guided projectiles. From the related research status, we can see that most of the current research on navigation technology of guided projectiles focuses on the application of GPS/INS integrated navigation technology to guided projectiles with low roll speed [8,15]. Although it has been successfully applied in many guided projectiles, the strapdown schemes adopted by GPS/GNSS are still only applicable to the projectiles with small roll speed, and these schemes have some fatal shortcomings, such as vulnerability to interference, poor reliability, and autonomy, and are always difficult to be accepted and popularized in the guidance transformation of rotating projectiles. Compared with the traditional strapdown inertial navigation system, the navigation accuracy of the GFSINS is lower and the error accumulation is faster, so the gyroscope-free strapdown inertial measurement system is still in the theoretical exploration stage [12,16]. In addition, due to the limitation of the GFSINS navigation principle and accelerometer manufacturing process, it is very difficult to continue to improve the navigation accuracy of the GFSINS [17,18]. Small caliber conventional rockets use servo motors to reverse rotation on the roll axis to keep the inertial measurement unit stationary or micro-rotation relative to the inertial space [19,20,21,22]. However, these single-axis stabilized inertial navigation platforms that utilize the motor to achieve the stability of the rolling shaft have complicated system structures and poor anti-overload performance due to the introduction of the servo motor, and are difficult to apply in conventional projectiles with high launch overloads.

In summary, most of the current navigation techniques of conventional rotating projectiles focus on solving the navigation guidance of non-rolling artillery or low-speed rolling projectiles. Among them, the advantages of the GFSINS are low cost and strong overload resistance, the disadvantage is that the error is large and the accuracy cannot be guaranteed. The advantage of integrated navigation and geomagnetic navigation systems is that errors do not accumulate over time, while the disadvantages are vulnerable to external interference, poor autonomy, and reliability. The advantage of the existing single-axis stabilized inertial navigation system is that it can isolate the influence of the projectile roll, while the disadvantage is the poor anti-overload ability, and it cannot be used in projectiles with large launching overloads.

In order to solve the problems encountered by the inertial measurement system in the application of small caliber, low cost, high overload, and high rotation conventional projectiles, by combining the characteristics of a platform inertial navigation system and strapdown inertial navigation system, based on a large number of theoretical analysis and practical experiments, the North University of China has proposed a passive semi-strapdown roll stabilized platform and carried out related research [23]. The passive semi-strapdown roll stabilized platform uses mechanical devices to isolate the micro inertial measurement unit (MIMU) from the projectile roll axis, and it has the function of “isolated rotation”. The platform is connected to the projectile through the bearings on the roll axis, which makes the MIMU stable in the roll axis direction of the projectile [24]. Because of the existence of bearings on the roll axis, the angular roll rate of the projectile directly acting on the platform is avoided, which reduces the requirement of the roll axis gyroscope range in the high-spin missile-borne environment. Thus, the purpose of calculating the attitude angle of the platform with high-precision was achieved and effectively measured the flight navigation parameters of the projectile [25].

Based on the passive semi-strapdown roll stabilized platform with unique structure, this paper focuses on the effect of the rolling isolation device on the stability of the platform. In order to make the semi-strapdown roll stabilized platform more stable and further reduce the angular swing rate of the platform, the bearing structure of the semi-strapdown roll stabilized platform was optimized and improved. This paper proposed a bearing nested structure that could reduce the frictional moment of the stabilized platform and used the existing friction moment calculation methods for reference. The dynamic modeling of the friction moment of double-stage and multi-stage bearing nested structures was carried out. The theoretical value of the friction moment of a bearing nested structure in a missile-borne environment was obtained, followed by a series of simulation analysis and ground semi-physical test verification. The simulation and test results showed that the proposed bearing nested structure had a better effect of isolating the projectile rolling angular rate, and thus improved the measurement accuracy of the inertial navigation system.

## 2. Working Principle of Passive Semi-Strapdown Roll Stabilized Platform 

### 2.1. Composition and Working Principle of the Platform

**Figure 1:**
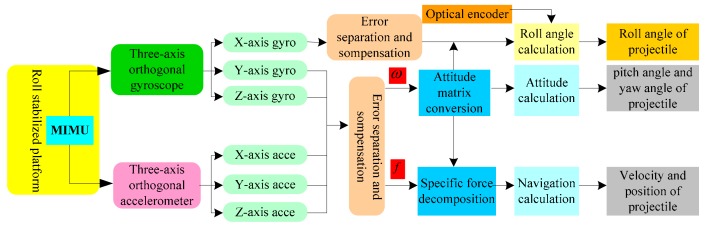
The working principle of the passive semi-strapdown roll stabilized platform.

The design of the semi-strapdown roll stabilized platform aims to solve the problem of the micro-inertial measurement system; low measurement accuracy due to the high axial speed of the projectile during the high-rotation navigation parameter measurement process. The semi-strapdown roll stabilized platform is a hybrid special mechanical device which combines the characteristics of a strapdown inertial measurement system and platform inertial measurement system. During the working process of the platform, the stabilized platform isolated the rolling motion of the projectile. Firstly, the motion parameters of the semi-strapdown roll stabilized platform were measured by MIMU, then, combined with the relative rotational speed information of the platform and the projectile measured by a photoelectric encoder, achieved calculation on the navigation solving circuit by the navigation algorithm, and finally, the flight navigation parameters of the projectile were obtained. Compared with the strapdown inertial navigation system, the semi-strapdown roll stabilized platform adopts the platform mounting method on the projectile roll axis [24]. Obviously, this solution is different from the installation mode in which the inertial device and the projectile are completely fixed in three axial directions in the strapdown inertial measurement system, and it is different from the installation mode of the three-axis completely isolated between the inertial device and the projectile in the platform inertial measurement system. Therefore, the algorithm arrangement is different from the strapdown algorithm and platform algorithm. The working principle of the passive semi-strapdown roll stabilized platform is shown in Figure 1. In the initial alignment, in addition to the initial attitude of the projectile, the initial attitude of the semi-strapdown roll stabilized platform on the roll axis was also loaded, then the attitude transformation matrix was established by combining the angular rate information of three orthogonally mounted gyroscopes, and combining the specific force information of three orthogonal accelerometers, obtaining the initial velocity, position, pitch angle, and yaw angle. In addition, the relative rotational angular rate information between the projectile body and the inner cylinder of the semi-strapdown roll stabilized platform was measured by a coded speed measuring device installed between the projectile and the inner cylinder of the semi-strapdown roll stabilized platform [25].

**Figure 2:**
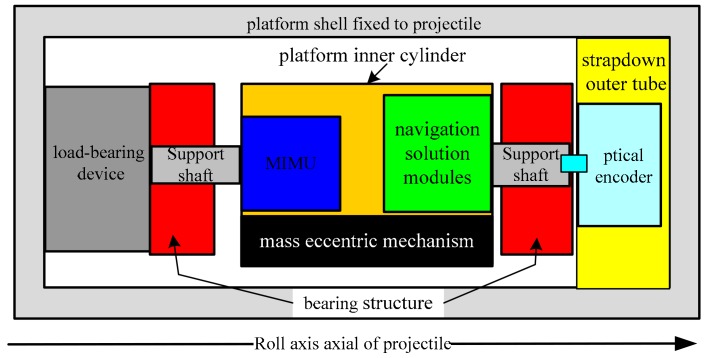
The structural composition diagram of the passive semi-strapdown roll stabilized platform.

The design of the passive semi-strapdown roll stabilized platform utilizes the principle of the compound pendulum under the action of gravity. The mass eccentric mechanism was adopted to make the inner cylinder of the platform get the restoring moment (restoring torque here refers to the gravity moment received by the mass eccentric mechanism) and keep the inner cylinder stable. The passive semi-strapdown roll stabilized platform could not only eliminate the adverse effects of the projectile roll on the MIMU, but also meet the requirements of volume and anti-overload performance. The mechanical structure of the platform mainly includes a platform shell, an inner cylinder with mass eccentric mechanism, bearing structure, load-bearing device, and strapdown outer tube. The specific composition is shown in Figure 2.

Inside the high spin projectile, the MIMU is mounted in the platform inner cylinder that is not attached to the projectile. The inner cylinder of the platform establishes a compound pendulum mass eccentric structure, and the front and rear sides use bearings to connect the external structure of the platform. During the operation of the platform, the inner cylinder of the platform maintains a small range of stable swing around the rolling shaft under the combined action of the gravity moment and the bearing friction moment. The angular rate isolation between the platform and the roll axis of the projectile is realized, and the strapdown connection between the platform and the projectile is maintained in the pitch and yaw directions. The load-bearing device was installed at the rear end of the projectile to ensure the normal operation of the bearing and the platform during the launching of the projectile. The MIMU and navigation solution module were installed in the inner cylinder of the platform [25,26]. The relative rotational speed information between the inner cylinder and the projectile was measured by a photoelectric encoder in the strapdown outer tube and transmitted to a navigation solution module inside the platform.

From the working principle of the passive semi-strapdown roll stabilized platform, it can be seen that the core is used to balance the friction moment caused by the rolling motion of the projectile to the platform by using a mass eccentric mechanism to obtain the gravity recovery moment, so as to stabilize the platform inner cylinder. The friction moment of the bearing is driven by the high-speed rolling motion of the projectile and platform gravity recovery moment, which are two main factors affecting platform motion, and which determine the stability effect of the platform inner cylinder motion. The smaller the bearing friction moment, the smaller the inner cylinder swing angular velocity of the platform, the better the effect of isolating the angular rate of the projectile. Therefore, in the case where the platform moment of inertia and mass eccentric mechanism have been determined, the design and optimization of the bearing device is the core content that could achieve stability and improve the measurement precision of the platform.

### 2.2. The Dynamic Model of Passive Semi-Strapdown Roll Stabilized Platform

**Figure 3:**
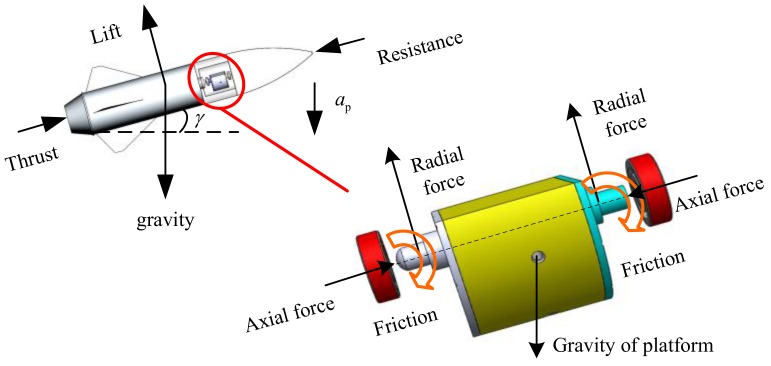
Schematic diagram of force of projectile flight and the platform inner cylinder.

**Figure 4:**
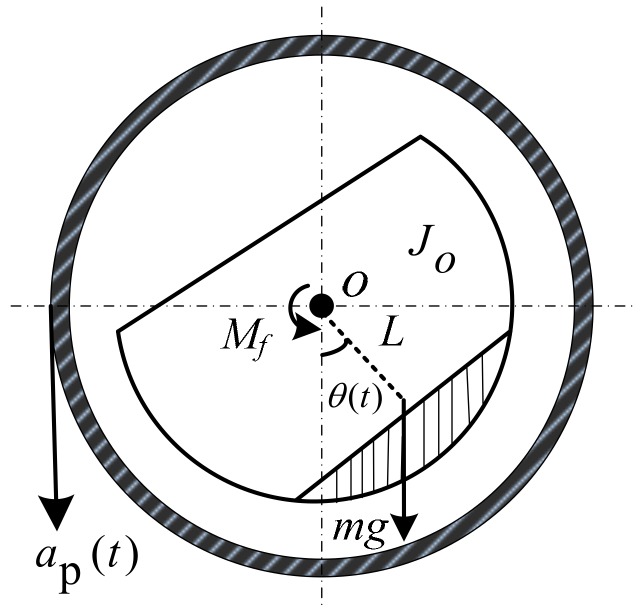
The mechanical model of the inner cylinder.

In order to analyze the influence of the bearing friction moment on the motion of the passive semi-strapdown roll stabilized platform, it is necessary to analyze the dynamic model of the platform. Since the guided projectile has characteristics of short range, small angle of attack, low-altitude flight, pitch, and yaw direction at a small angular rate, in the mechanical analysis of the projectile, the main consideration is the effect of lift, gravity, thrust, and resistance on the projectile in flight. It is assumed that the angle of attack is small when the projectile is flying. During the flight of the projectile, the magnitude and direction of gravity can be considered constant, and the direction of the resistance coincides with the longitudinal axis of the projectile. The direction of gravity is vertically downward, the direction of the lift is perpendicular to the upward direction of the projectile, the direction of the resistance is opposite to the direction of flight of the projectile, and the direction of the thrust is the same as the direction of flight of the projectile. In addition, the acceleration of the projectile in the vertical direction is $a_{p}$. The magnitude and direction of $a_{p}$ is determined by the resultant force of gravity and lift exerted on the projectile. The force and movement of the projectile during flight determine the force of the inner cylinder of the passive semi-strapdown roll stabilized platform installed inside. During the flight of the projectile, the inner cylinder of the platform is subjected to friction, gravity, radial force, and axial force. The force acted on the projectile and inner cylinder of the passive semi-strapdown roll stabilized platform is shown in Figure 3. According to the stress situation of the platform inner cylinder and the problems to be solved, the mechanical model of the inner cylinder was established, as shown in Figure 4.

When the projectile is rotating at a high speed, the bearing is driven to roll, and the bearing exerts frictional force on the inner cylinder of the platform, which makes the inner cylinder rotate around the roll axis. When the centroid of the inner cylinder rotates away from the equilibrium position in the vertical direction, the component of gravity will provide the restoring moment (because the inner cylinder of the platform and the projectile have the same acceleration in the vertical direction, the restoring moment at this time is provided by the equivalent gravity, which here refers to the resultant force of gravity and lift on the platform inner cylinder in the vertical direction during the flight of the projectile).

According to the mechanical model shown in Figure 4, and by combining the dynamic principle of the compound pendulum, the dynamic equation of the inner cylinder is established. The resultant moment M of the inner cylinder is equal to the vector sum of the bearing friction moment $M_{f}$ and the equivalent gravity moment $M_{g}$, i.e.,

(1) $$M=M_{f}-M_{g}.$$

The equivalent gravity moment of the inner cylinder can be expressed as follows:

(2) $$M_{g}=m\left[g-a_{P}(t)\right]\cdot L\sin\theta (t)\cos\gamma (t),$$

where m is the mass of the inner cylinder; $a_{p}$ is the acceleration of the projectile in the vertical direction, L is the equivalent pendulum length of the inner cylinder, θ(t) is the swing angle of the inner cylinder around the roll axis of the projectile, and γ(t) is the pitch angle of the projectile during flight.

According to the theorem of angular momentum, the sum of the algebraic components of the external moment along the fixed axis is equal to the product of the moment of inertia and angular acceleration of the axis. Therefore, the dynamic equation of the inner cylinder on the roll axis of the projectile is as follows:

(3) $$M_{f}-m\left[g-a_{p}(t)\right]\cdot L\sin\theta (t)\cos\gamma (t)=J_{o}\frac{d^{2}\theta (t)}{d^{2}t}$$

where $J_{o}$ is the moment of inertia of the inner cylinder relative to the centroid.

From the above dynamic analysis, it can be seen that the friction of the bearing device directly affects the swing angular rate of the platform inner cylinder, thereby affecting its stability.

## 3. Improvement Principle of Bearing Device

**Figure 5:**
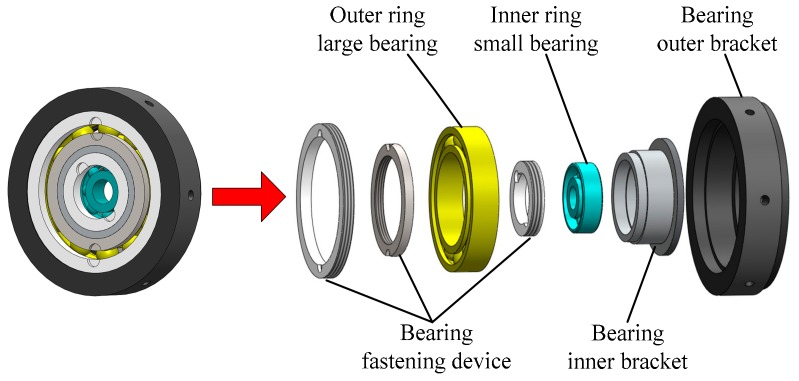
Overall schematic diagram of bearing nested structure.

According to the missile-borne application environment of the passive semi-strapdown roll stabilized platform, the bearing device not only bears the axial and radial loads, but also supports the inner cylinder and isolates the rolling motion of the projectile. This situation requires that during the operation of the platform, the friction moment generated by the bearing should be as small as possible to ensure the small swing amplitude of the platform. For the traditional single-bearing device, the problem of friction increases or even poor rotation can easily occur during the operation, which directly affects the stability performance of the platform and makes the angular velocity of the platform inner cylinder increase, which makes it difficult to meet the stability requirements. In view of the above application requirements, the multi-stage bearing nested structure based on bearing combination is proposed. The structure integrates a plurality of bearings in a longitudinal direction, adopts a “multi-stage isolation, indirect drive” friction moment generation method, effectively reduces the bearing friction moment, and utilizes a plurality of bearings to improve the axial carrying capacity of the bearing. Taking the double-stage bearing nested structure as an example to illustrate the optimal design of the bearing nested structure. The overall schematic diagram of the bearing nested structure is shown in Figure 5. The bearing nested structure is composed of a bearing fastening device, large bearing, small bearing, bearing inner bracket, and outer bracket. Among them, a bearing fastening device is used to fasten inner and outer two-stage bearings, a large bearing connects a small bearing and a bearing outer bracket to reduce the rotation speed, a small bearing connects a large bearing and the platform inner cylinder to further reduce the rotation speed and friction moment, a bearing inner bracket and outer bracket are fixed to the inner and outer bearings respectively to keep the two-stage bearings coaxial, and the outer bearing bracket is fixed behind the platform by screw fastening to ensure that the bearing nested structure and platform are coaxial.

Assembly method of large bearing nested small bearing. The outer ring of the large bearing is fixed to the platform shell through the bracket, the inner ring of the small bearing is connected with the inner cylinder mounting shaft. The bearing brackets are designed with bosses to ensure the positioning and installation of the bearing and coaxiality with the system. During the operation of the platform, the large bearing isolates the high-speed rolling motion of the external projectile, and the friction moment generated by it drives the small bearing to rotate. The small bearing generates a small friction moment which is then transmitted to the inner cylinder of the platform. Here, the friction moment acting on the platform inner cylinder is only the moment formed after attenuation of the two-stage bearings. The bearing nested structure greatly reduces the influence of the friction moment and improves the motion stability of the platform.

## 4. Calculation Model of Friction Moment in Bearing

### 4.1. Calculation of the Traditional Signal Bearing Friction Moment 

In general, according to the type, structure, size, and conditions of use of the bearing, the bearing friction moment can be approximated by the following formula [27,28]:

(4) $$M_{a}=\frac{1}{2}\mu dP,$$

where μ is the friction coefficient of the bearing, d is the inner diameter of the bearing, and P is the equivalent dynamic load of the bearing. However, this estimation method can only calculate the average friction moment under the condition of good lubrication and medium speed rotation. This method is not suitable for the case of high-speed rotation, and the estimation is very inaccurate.

The friction moment of the rolling bearing depends on the type, size, load, speed, lubrication, and sealing. If the total friction moment of the rolling bearing is to be calculated accurately, the following factors must be considered: the influence of the rolling friction moment, lean oil backfill and cut-in fever effect, sliding friction moment and its influence on lubrication quality, friction torque of sealant, friction moment caused by resistance loss, agitation, and splash. The moment that causes the bearing to operate, which is usually caused by the sum of rolling friction, sliding friction, and lubricant friction, is called the friction moment.

Harris considered six factors in rolling bearing friction: the elastic hysteresis of materials in rolling materials, rolling body-raceway contact slip caused by the geometry of the contact surface, sliding caused by deformation of the contact body, sliding between the cage and rolling element, lubricant drag friction of lubricant on the rolling element and cage, and friction between the cage and guide surface. Harris’s empirical formula is [29]:

(5) $$M_{h}=f_{1}F_{b}d_{m}+1.42\times 10^{-5}{\left(\upsilon_{0}n\right)}^{2/3}d_{m}{}^{3},$$

where $M_{h}$ is the total friction moment, $f_{1}$ is the coefficient dependent on the bearing structure and the relative acting load of the bearing, $F_{b}$ is the equivalent load, $d_{m}$ is the diameter of the rolling bearing, $\upsilon_{0}$ is the motion viscosity of the lubricating oil, and *n* is the speed of bearing.

This method takes into account the bearing load factor and belongs to the common friction moment estimation method. However, as an important factor affecting the motion of the platform, the bearing friction moment directly affects the analysis result of the platform. Therefore, it is necessary to consider various factors affecting the friction moment and use a more accurate calculation method.

According to the fundamental cause of bearing friction, the SKF (Svenska Kullager Fabriken) company has given a more accurate method to calculate the friction moment of rolling bearings [27]:

(6) $$M=\phi_{r}\phi_{i}M_{\mathrm{rr}}+M_{\mathrm{sl}}+M_{\mathrm{seal}}+M_{\mathrm{drag}},$$

where $\phi_{r}$ is the lean oil backfill reduction factor, which can be determined by the following formula:

$$\phi_{r}=\frac{1}{eK_{r}\upsilon n\left(d+D\right)\sqrt{\frac{K_{Z}}{2(D-d)}}},$$

where *n* is the bearing speed, υ is the moving viscosity of lubricant at the working temperature, $K_{r}$ is the lean oil backfill constant, $K_{Z}$ is the geometric constant determined according to the bearing type, *D* is the bearing outer diameter, and *d* is the bearing inner diameter.

$\phi_{i}$ is the cut-in heat reduction factor, which can be determined by the following formula:

$$\phi_{i}=\frac{1}{1+1.84\times 10^{-9}{\left(nd_{m}\right)}^{1.28}\upsilon^{0.64}},$$

where $d_{m}=\frac{D+d}{2}$ is the pitch diameter of the bearing.

$M_{\mathrm{seal}}$ is the friction moment of seals, because the passive semi-strapdown roll stabilized platform is a sealing system, the open type bearing is chosen, so this item is not calculated here. $M_{\mathrm{drag}}$ is the friction moment caused by drag loss, eddy current, and splash (for grease lubrication $M_{\mathrm{drag}}=0$), since the bearing in this paper is grease lubricated, there is $M_{\mathrm{drag}}=0$.

$M_{\mathrm{rr}}=G_{\mathrm{rr}}{\left(\upsilon n\right)}^{0.6}$ is the rolling friction moment, where $G_{\mathrm{rr}}$ is the rolling friction variable determined by bearing type.

$M_{\mathrm{sl}}=G_{\mathrm{sl}}\mu_{\mathrm{sl}}$ is the sliding friction moment, where $G_{\mathrm{sl}}$ is the sliding friction variable determined by bearing type, and $\mu_{\mathrm{sl}}$ is the sliding friction coefficient [28,29]. 

The calculation method of $G_{\mathrm{rr}}$ and $G_{\mathrm{sl}}$ are shown in Table 1.

The deep groove ball bearing which can bear radial load as well as some axial load and composed load is one type of non-separable bearing as well as the most typical structure of rolling bearing. Angular contact ball bearings have inner and outer ring raceways that are displaced relative to each other in the direction of the bearing axis, meaning that these bearings are designed to accommodate combined loads [30,31,32,33,34,35]. $F_{r}$ and $F_{a}$ are external radial and axial loads, $C_{0}$ is the rated static load of the bearing, and $S_{1}$, $R_{2}$, $R_{3}$, $S_{1}$, $S_{2}$, and $S_{3}$ are geometric constants of the bearing.

From the above analysis, the traditional single bearing friction moment calculation (1) can be simplified to the following results [27]:

(7) $$M=\phi_{r}\phi_{i}M_{\mathrm{rr}}+M_{\mathrm{sl}}=\phi_{r}\phi_{i}G_{\mathrm{rr}}{\left(\upsilon n\right)}^{0.6}+G_{\mathrm{sl}}\mu_{\mathrm{sl}}.$$

Obviously, the calculation method of SKF is more accurate. Therefore, when calculating the bearing friction moment, the SKF bearing friction moment (7) is selected.

### 4.2. Establishment of a Calculation Model for the Friction Moment of a Bearing Nested Structure

**Figure 6:**
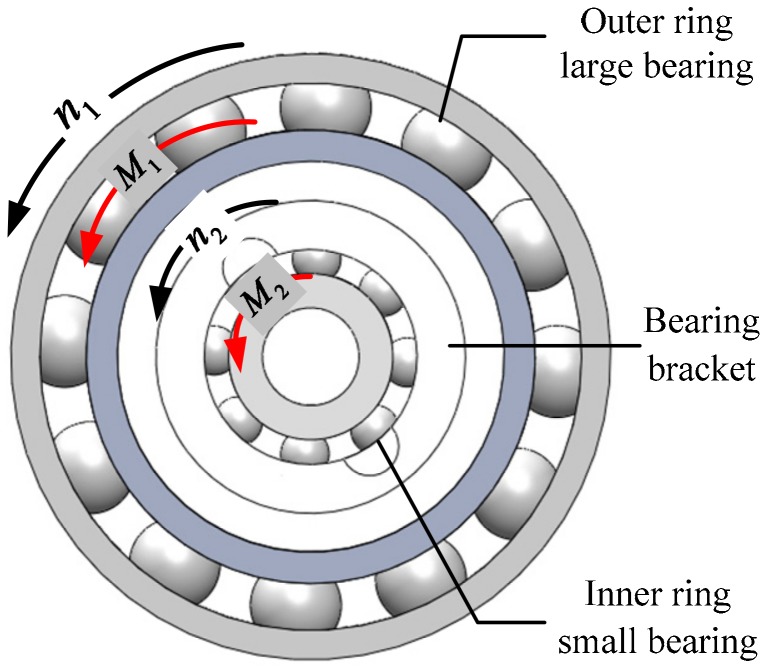
Friction moment transfer principle of a two-stage bearing nested structure.

The double-stage bearing nested structure adopts the mode of “two-stage isolation, indirect drive” to generate a friction moment. Installed by a large bearing nesting a small bearing, the large bearing is fixedly connected with a platform shell. Driven by the high-speed rolling motion of the projectile body, the friction moment is generated to drive the inner ring small bearing to run, which makes the small bearing produce a smaller friction moment and then transmit it to the platform inner cylinder. In this case, the friction moment on the inner cylinder of the platform is only the moment formed after attenuation of the two-stage bearing, which reduces the friction moment generated by the bearing directly driven by the external projectile. The principle of friction moment generation and transmission is shown in Figure 6.

According to the aforementioned transfer principle of a friction moment, it was supposed that the external projectile rotates at high speed $n_{1}$ that drives the large bearing outer ring to rotate, and the friction moment produced is $M_{1}$. The bearing bracket is used to connect the large bearing inner ring with the small bearing outer ring. The friction moment $M_{1}$ drives the bearing bracket to rotate at $n_{2}$, and then drives the small bearing to rotate. The resulted friction moment is $M_{2}$, here, $M_{2}$ is the final output friction moment of the bearing nested structure, and as the driving moment of the platform inner cylinder, it is transmitted to the platform inner cylinder.

According to the calculation method of the single bearing friction moment of (7), the expression of the friction moment produced by the large bearing is as follows:

(8) $$M_{1}=\phi_{r1}\phi_{i1}M_{\mathrm{rr}1}+M_{\mathrm{sl}1}=\phi_{r1}\phi_{i1}G_{\mathrm{rr}1}{\left[\upsilon \left(n_{1}-n_{2}\right)\right]}^{0.6}+G_{\mathrm{sl}1}\mu_{\mathrm{sl}1}.$$

Because the swing angular velocity of the platform inner cylinder driven by the bearing nested structure is small and can be neglected relative to the rotational speed of the small bearing, the expression of the friction moment produced by the small bearing is as follows:

(9) $$M_{2}=\phi_{r2}\phi_{i2}M_{\mathrm{rr}2}+M_{\mathrm{sl}2}=\phi_{r2}\phi_{i2}G_{\mathrm{rr}2}{\left[\left(\upsilon n_{2}\right)\right]}^{0.6}+G_{\mathrm{sl}2}\mu_{\mathrm{sl}2}.$$

As a bearing bracket connecting two bearings, the friction moment produced by the two bearings acts on it. The friction moment $M_{1}$ generated by the large bearing drives the movement of the bearing bracket, and the direction of friction moment $M_{1}$ is the same as that of the bracket movement. The small bearing is driven by the bracket, the friction moment $M_{2}$ generated hinders movement of the bracket, and the direction of the friction moment $M_{2}$ is opposite to the direction of bracket movement.

Therefore, the total moment borne by the bearing bracket is the vector sum of the friction moment generated by the two bearings, that is:

(10) $$M=M_{1}-M_{2}.$$

The bearing bracket rotates at a speed of $n_{2}$, which can be equivalent to a rigid body that rotates along the center axis of the projectile. According to the rigid body rotation law, when a rigid body rotates on a fixed axis, its external moment is equal to the product of the moment of inertia of the rigid body and angular acceleration, that is:

(11) $$M=J_{b}\frac{dn_{2}}{dt},$$

where the moment of inertia of the bearing bracket is $J_{b}=\frac{1}{2}m_{b}\left(r_{1}^{2}+r_{2}^{2}\right)$. $m_{b}$ is the quality of the bearing bracket, $r_{1}$ and $r_{2}$ are the inner and outer diameters of the bracket respectively, and the size is determined by the bearing model.

Combining (8)–(11), the final calculation model of the friction moment of the double-stage bearing nested structure is obtained as follows:

(12) $$\phi_{r1}\phi_{i1}G_{\mathrm{rr}1}{\left[\upsilon (n_{1}-n_{2})\right]}^{0.6}+G_{\mathrm{sl}1}\mu_{\mathrm{sl}1}-\phi_{r2}\phi_{i2}G_{\mathrm{rr}2}{\left(\upsilon n_{2}\right)}^{0.6}+G_{\mathrm{sl}2}\mu_{\mathrm{sl}2}=J_{b}\frac{dn_{2}}{dt}.$$

Equation (12) is the transfer model of the friction moment of the double-stage bearing nested structure, which represents the transfer relationship of the friction moment of two bearings in the bearing nested structure when they are running, and (9) is the calculation equation of the final output friction moment of the double-stage bearing nested structure, the friction moment is transmitted to the platform inner cylinder and affects the motion of the platform.

According to the modeling method of the friction moment of the double-stage bearing nested structure mentioned above, for the multi-stage bearing nested structure, the following friction moment dynamic model can be obtained:

(13) $$\left\{\begin{array}{c} M_{1}=\phi_{r1}\phi_{i1}G_{\mathrm{rr}1}{\left[\upsilon (n_{1}-n_{2})\right]}^{0.6}+G_{\mathrm{sl}1}\mu_{\mathrm{sl}1}\\ M_{1}-M_{2}=J_{1}\frac{d(n_{2}-n_{3})}{dt}\\ M_{2}-M_{3}=J_{2}\frac{d(n_{3}-n_{4})}{dt}\\ \vdots \\ M_{n-1}-M_{n}=J_{n-1}\frac{dn_{n}}{dt}\\ M_{n}=\phi_{rn}\phi_{in}G_{\mathrm{rr}n}{\left(\upsilon n_{n}\right)}^{0.6}+G_{\mathrm{sl}n}\mu_{\mathrm{sl}n} \end{array}\right.,$$

where $n_{i}$(i=1,2,⋯,n) is the rotational speed of the ith bearing, and $J_{j}$(j=1,2,⋯n−1) is the rotational inertia of the ith bearing bracket.

The final calculation model of the friction moment of the n-stage bearing nested structure is obtained from the above:

(14) $$\begin{array}{l} \phi_{r1}\phi_{i1}G_{\mathrm{rr}1}{\left[\upsilon (n_{1}-n_{2})\right]}^{0.6}+G_{\mathrm{sl}1}\mu_{\mathrm{sl}1}-\phi_{rn}\phi_{in}G_{\mathrm{rr}n}{\left(\upsilon n_{n}\right)}^{0.6}+G_{\mathrm{sl}n}\mu_{\mathrm{sl}n}\\ =J_{1}\frac{d(n_{2}-n_{3})}{dt}+J_{2}\frac{d(n_{3}-n_{4})}{dt}+\ldots +J_{n-2}\frac{d(n_{n-1}-n_{n})}{dt}+J_{n-1}\frac{dn_{n}}{dt} \end{array}$$

(15) $$M_{n}=\phi_{rn}\phi_{in}G_{\mathrm{rr}n}{\left(\upsilon n_{n}\right)}^{0.6}+G_{\mathrm{sl}n}\mu_{\mathrm{sl}n}$$

Equation (14) is the transfer model of the friction moment of the n-stage bearing nested structure, and (15) is the calculation equation of the final output friction moment of the n-stage bearing nested structure.

### 4.3. Bearing Type Selection and Relevant Parameters

In the past, the passive semi-strapdown roll stabilized platform adopted a single bearing structure that is SKF6200 deep groove ball bearing.. For the two-stage bearing nested structure, according to the application environment and load-bearing type of the passive semi-strapdown roll stabilized platform, combined with the actual internal dimensions and loads of the passive semi-strapdown roll stabilized platform for a rotating projectile, an SKF7008AC angular contact ball bearing was selected for the large outer ring bearing to withstand radial and axial combined loads during external ballistic flight, an SKF6200 deep groove ball bearing was selected for the inner ring small bearing, which could reduce the friction moment of the system as much as possible while bearing residual load, so as to realize the complementary advantages of the bearing application. Both kinds of ball bearings belong to small precision bearings, and the change of friction moment caused by size change is relatively small. The relevant parameters of the two kinds of bearings are shown in Table 2.

Considering the narrow available space of the projectile, the stability of the bearing nested structure, and the coaxiality of the system, the method of two-stage bearing nested structure was finally adopted in this paper. The following series of experiments were verified by taking the double-stage bearing nested structure as an example.

## 5. Simulation Verification

### 5.1. Axial Reliability Verification of the Bearing Nested Structure

When the passive semi-strapdown roll stabilized platform works in the missile-borne environment, the large overload such as launch overload is mainly borne by the load-bearing device of the platform. In the inertial flight phase of the projectile, the axial load is mainly borne by the bearing device. In order to verify the axial reliability of the bearing nested structure, the finite element mechanical simulation of the bearing nested structure was carried out.

The bearing structure is mainly composed of a bearing outer ring, bearing inner ring, rolling element, and bracket. Because of the complex shape of the bracket, the bracket has little influence on the bearing contact characteristics analysis during the actual contact of the bearing. Therefore, the bearing bracket is simplified, and the geometry model is simplified as follows: (1) neglecting the influence of chamfer of the rolling bearing on internal stress of the bearing; (2) ignoring the influence of axial clearance of the bearing and lubrication film on the bearing; (3) ignoring the non-linearity of the material.

The following conditions were set for finite element analysis: the bearing structure has an outer ring bracket, and the bearing structure is completely fixed in the platform by screw fastening, so the boundary condition can be set to set the bearing outer ring bracket as a fixed support constraint; according to the actual missile-borne application environment, the maximum axial overload of the projectile during the inertial flight phase after launch is generally less than 10*g*, so in the inertial flight phase after the projectile is launched, the maximum axial loads borne by the bearing structure does not exceed 10*g*. When performing the simulation, a loading constraint of 10*g* is applied to the axial force receiving faces of the two bearing structures respectively (that is, the loading constraint is to apply 10*g* overload respectively in the z direction; all the components of the bearing structure are made of GCr15 bearing steel, and the corresponding material parameters and properties are shown in Table 3.

**Figure 7:**
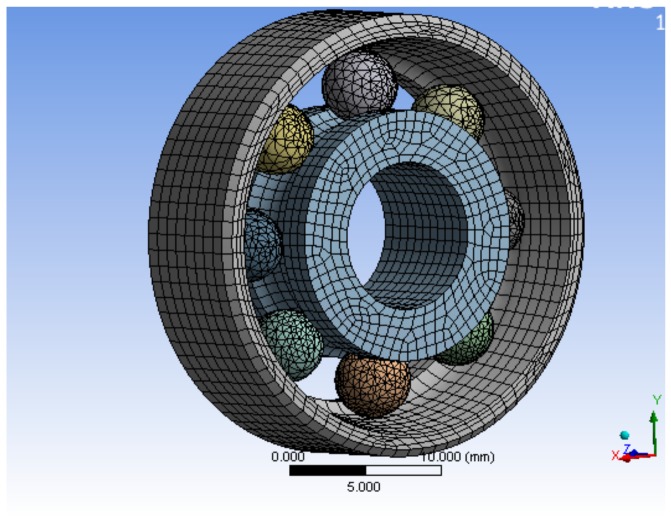
Model meshing of a single bearing structure.

**Figure 8:**
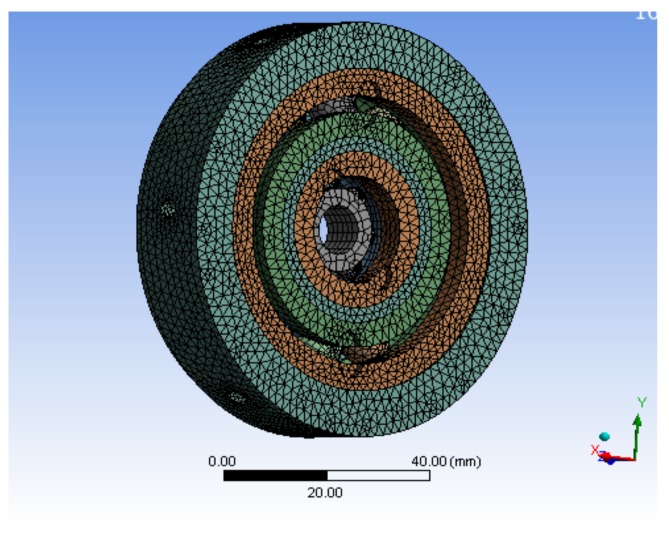
Model meshing of a bearing nested structure.

The bearing structure has a more cambered structure and spherical structure, so in the process of meshing, SOLID164 body element was selected as the finite element, and mesh generation was carried out by combining scan meshing, map meshing, and free meshing, according to the complexity of the bearing component structure. The inner and outer rings and the rolling elements of the bearing adopted a hexahedral element, and the brackets adopted a tetrahedral element, which was completed by using reasonable precision level mesh, the simulation model and mesh of single bearing structure, and the bearing nested structure as shown in Figure 7 and Figure 8. The processing of the joint surface was connected by the ADD or ULLTE Boolean operation command, and the influence occurs in the form of boundary conditions between components. The analysis results included the adverse effects caused by ignoring the joint surface, and the analysis results included the adverse effects caused by neglecting the interface. It can be seen from the subsequent analysis results that the results can converge well under the method of mesh generation and can mesh the model with a reasonable division accuracy level.

**Figure 9:**
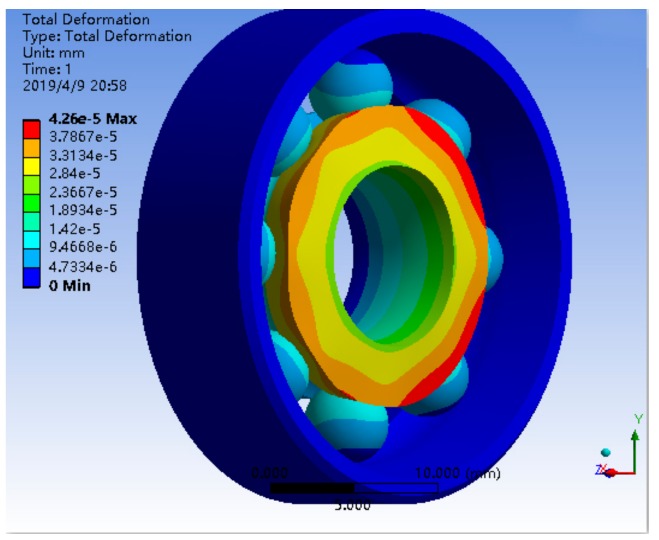
The deformation distribution of a single bearing structure under 10*g*.

**Figure 10:**
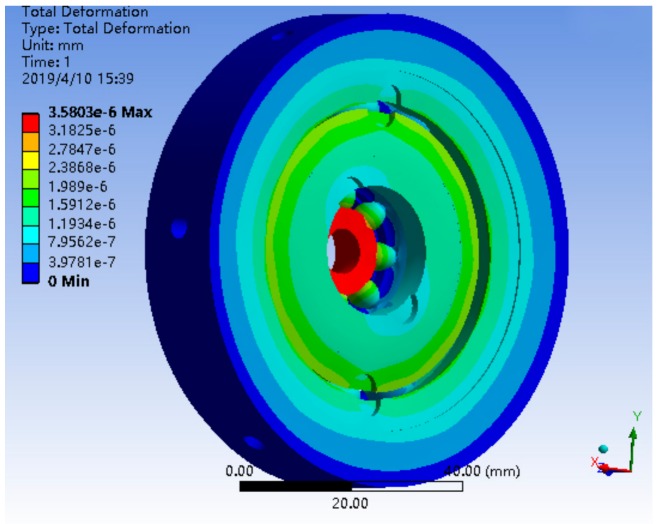
The deformation distribution of a bearing nested structure under 10*g*.

**Figure 11:**
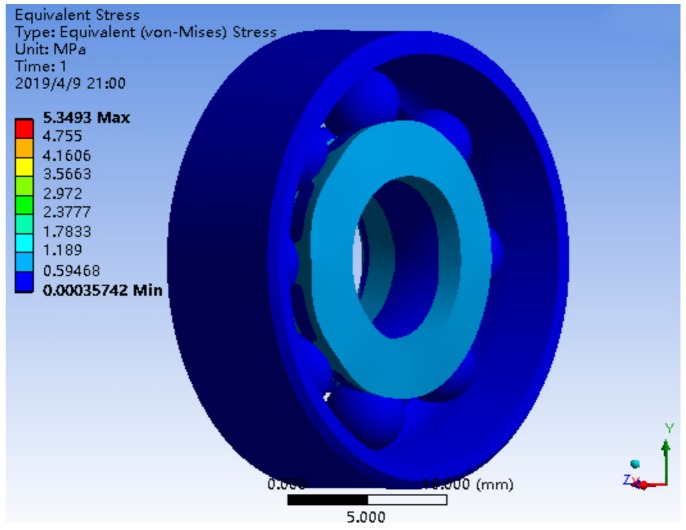
The stress distribution of a single bearing structure under 10*g*.

**Figure 12:**
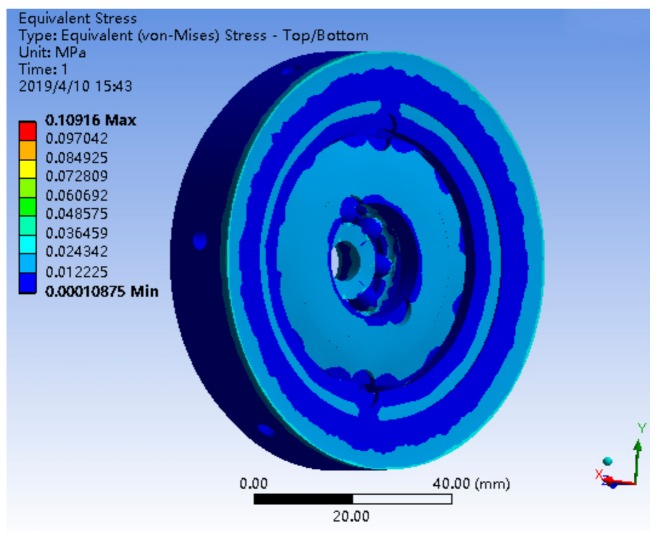
The stress distribution of a bearing nested structure under 10*g*.

The deformation distribution of a single bearing structure and bearing nested structure is shown in Figure 9 and Figure 10. The stress distribution is shown in Figure 11 and Figure 12.

The maximum deformation of a single bearing structure under a 10*g* axial load is 4.26 × 10^−5^ mm, and that of a bearing nested structure under a 10*g* axial load is 3.5803 × 10^−6^ mm. Obviously, under the same axial load, the maximum deformation of the bearing nested structure is reduced by an order of magnitude compared with that of the single bearing structure.

The maximum stress of a single bearing structure under a 10*g* axial load is 5.3493 MPa, and that of a bearing nested structure under a 10*g* axial load is 0.10916 MPa. Obviously, under the same axial load, the maximum stress of the bearing nested structure is reduced by an order of magnitude than that of the single bearing structure.

This simulation analysis is mainly to verify the axial bearing capacity of the bearing nested structure, and the simulation model is appropriately simplified. Therefore, the simulation analysis mainly considered the axial load and almost did not consider other factors. Through the above simulation analysis, it can be seen that under the same axial load, the bearing nested structure has better axial reliability than the single bearing structure. The introduction of the bearing nested structure makes the deformation and stress of the bearing device smaller, and makes the operation of the bearing device more reliable.

### 5.2. Calculation of the Theoretical Frictional Moment of a Bearing Nested Structure

The magnitude of bearing friction moment is related to factors such as rotational speed and load in the application environment. The calculation of the bearing friction moment should be based on the practical application environment of the platform. The passive semi-strapdown roll stabilized platform took a gun-launched missile as the missile test platform, and the maximum speed of the projectile was 15 r/s (5400°/s). Relevant parameters of the platform inner cylinder are shown in Table 4.

During the projectile flight, the radial support force of the bearing structure was the platform gravity, and the maximum axial force borne by the bearing structure did not exceed 98 N, i.e., $F_{a\max}=98\;N$.

According to the above missile-borne application environment, the working conditions of the bearing structure inside the platform were as follows: the outer ring of the large bearing ran at a speed of 900 r/min; the actual radial load was $F_{r}=9.8\;N$, the axial load was $F_{a}=98\;N$, and lubricating with mineral grease, the kinematic viscosity was taken as $\upsilon =68\;{\mathrm{mm}}^{2}/s$, $\mu_{\mathrm{sl}1}=\mu_{\mathrm{sl}2}=0.05$. The bearing worked in a confined space, and an open type bearing without sealing rings was selected. The rotational inertia of the bearing bracket was $J_{b}=312.5\;\mathrm{kg}\times {\mathrm{mm}}^{2}$.

**Figure 13:**
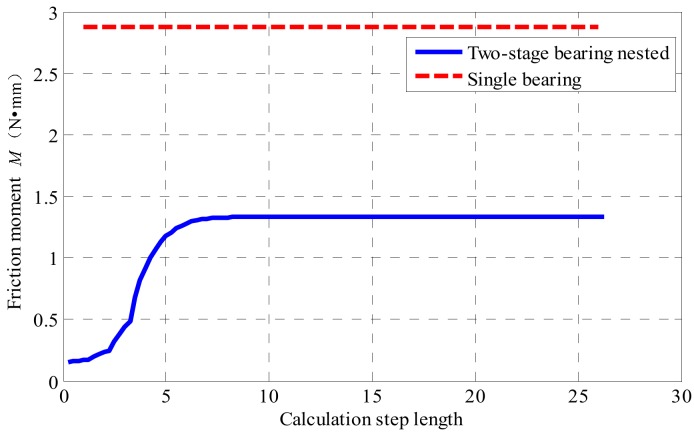
Friction moment of two bearing structures under 900 r/min.

According to the formula of the bearing friction variable in Table 1 and the basic parameters of the bearing shown in Table 2, the rolling friction moment coefficient and sliding friction moment coefficient of two kinds of bearings are calculated. Under the same load and speed environmental conditions, the friction moment of a single bearing structure and bearing nested structure are calculated respectively. For the single bearing (deep groove ball bearing 6200) as the connecting device of the platform, the relevant parameters are brought into (6), and the friction moment produced by the single bearing structure is M=2.88 N. For the bearing nested structure, the relevant parameters are substituted into the friction moment transfer model of the bearing nested structure (Equation (14)) and the friction moment output model of the bearing nested structure (Equation (15)). Equation (14) is a first-order non-linear differential equation, this kind of equation is difficult to find a general solution, so the classical fourth-order Runge-Kutta method is used to solve the formula. The function is solved by the adaptive variable step method in the operation process. The friction moment produced by the two bearing structures is shown in Figure 13.

It can be seen from Figure 11 that when the outer ring rotation speed is 900 r/min, the outer ring of the bearing nested structure drives the inner ring small bearing to run, and the output moment torque increases continuously from the start-up friction moment, and finally remains stable at the maximum value of 1.33 N·mm. The maximum frictional moment generated by the bearing nested structure is 1.33 N·mm, and the friction moment generated by the single bearing structure is 2.88 N·mm. The friction moment generated by the bearing nested structure is obviously less than that generated by the single bearing, and the output stability value of the friction moment is 47% that of a single bearing.

**Figure 14:**
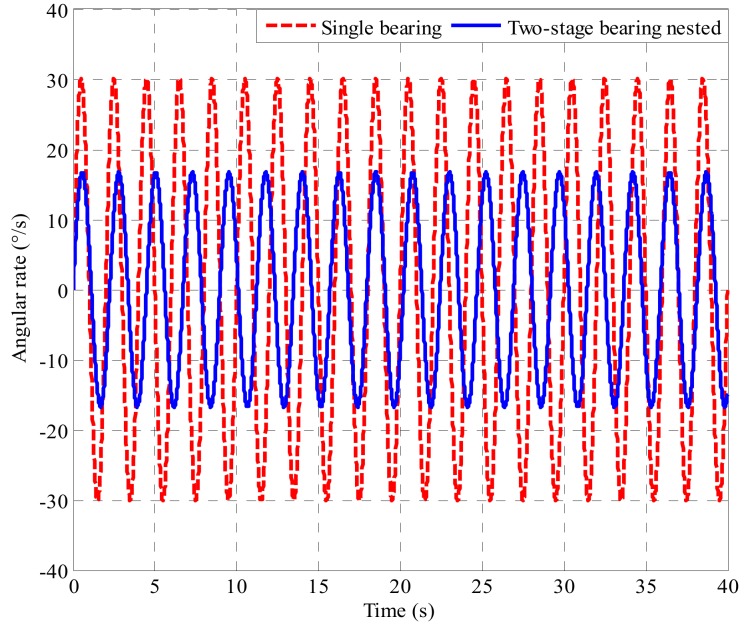
Simulation of the platform inner cylinder swing angular rate based on the single bearing and two-stage bearing nested structure under 900 r/min.

Under the condition that the rotary inertia and mass of the platform inner cylinder of the structure are constant, the friction moment generated by the single bearing structure and the friction moment generated by the bearing nested structure are substituted into (3) respectively, and combined with the fourth-order Runge-Kutta method to solve the angular rate of the inner cylinder. The amplitude of the angular swing rate of the platform inner cylinder based on the bearing nested structure is 50% of that of the platform inner cylinder based on the single bearing structure (as shown in Figure 14). The parameters needed in the calculation are shown in Table 4.

Through theoretical calculation and simulation analysis, from results gathered in Figure 14, it can be seen that the effect of the bearing nested structure separating the roll of the projectile is obviously superior to the single bearing structure, and the proposed method of the bearing nested method cannot be only to reduce the angular rate from around 30 °/s to around 15 °/s, but also can reduce the swinging frequency of the inner cylinder. This means that under the proposed improved method, the passive semi-strapdown roll stabilized platform can use sensors with smaller range and higher precision, thus, the accuracy of the attitude and position measurement can be improved.

## 6. Test Verification

### 6.1. Impact Test

**Figure 15:**
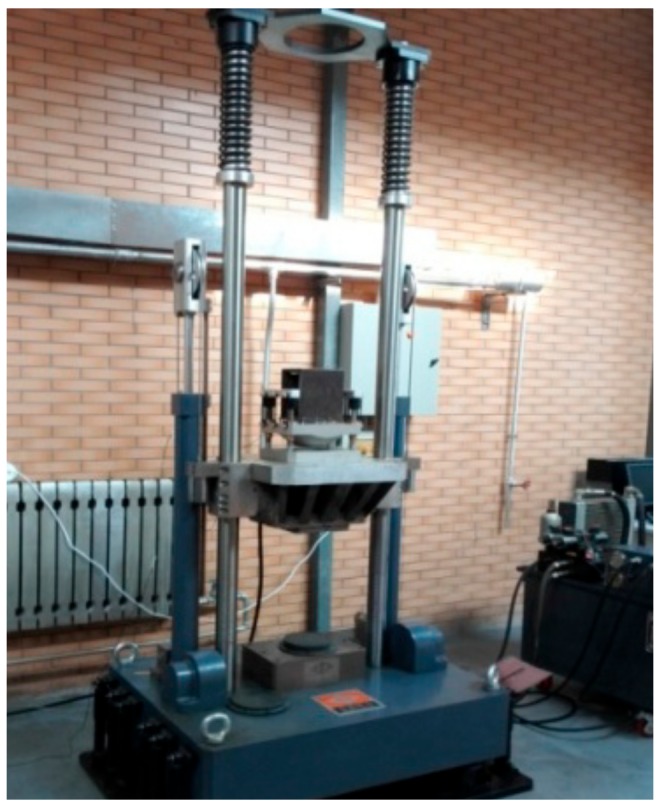
Impact test site.

Before the vehicle experiment, in order to test the axial bearing capacity of the bearing nested structure, the impact test of the single bearing and the bearing nesting structure were carried out respectively. The impact test site is shown in Figure 15.

**Figure 16:**
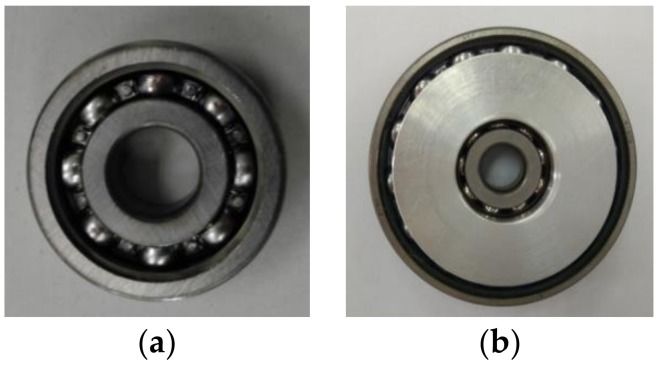
(**a**) The state of a single bearing after the impact test. (**b**) The state of the bearing nested structure after the impact test.

The single bearing and the bearing nested structure are fixed on the hydraulic impact test bench respectively, and the hydraulic impact test bench is adjusted to the falling height corresponding to the acceleration of 10*g*. The impact tests of two kinds of bearings were carried out under the same overload environment. The states of the two kinds of bearing structures are shown in Figure 16.

After the impact test, the bearing nested structure had hardly produced any plastic deformation, could still operate normally, and there was not any stuck condition. The impact test shows that the bearing nested structure had good axial load-bearing performance under the acceleration of 10*g*, which indicates that the overload of the projectile inertial flight phase does not affect the normal operation of the bearing nested structure.

### 6.2. Vehicle Test

**Figure 17:**
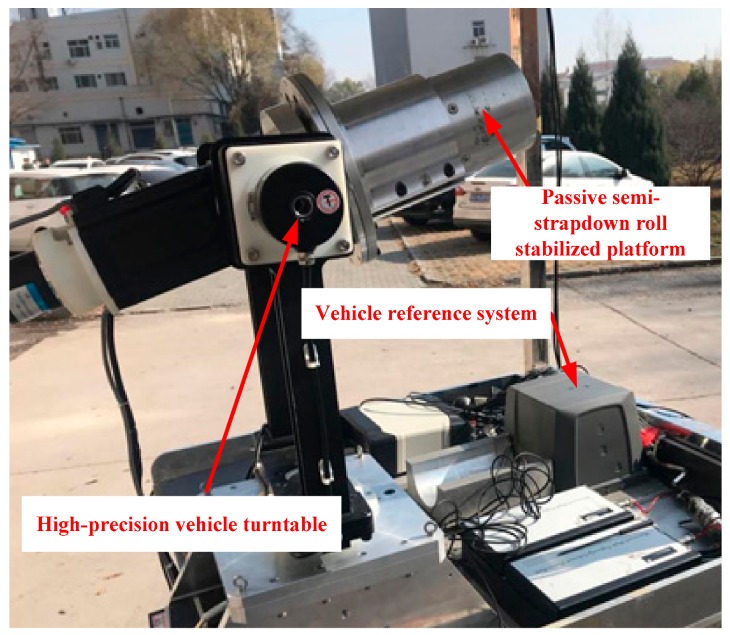
The vehicle test platform.

In order to verify the effect of the introduction of the bearing nested structure on improving navigation accuracy of the passive semi-strapdown roll stabilized platform, and to simulate the corresponding live ammunition application environment, the ground semi-physical dynamic car test was carried out, as shown in Figure 17, which was the vehicle test platform. The test platform was equipped with a high-precision vehicle turntable, which could simulate the real-time high-speed rotation motion of the projectile after the high-speed rotating projectile is launched on the ground, and provided high-precision roll angle feedback information in real time (attitude determination accuracy of the rolling angle of the vehicle-mounted high-precision turntable: 0.05°). The vehicle test platform was also equipped with SPAN-LCI (system attitude measurement accuracy: pitch angle of 0.008°, roll angle of 0.008°, yaw angle of 0.023°), a high-precision dynamic attitude determination and positioning system from NovAtel, Canada, which provided a real-time navigation parameter reference for the passive semi-strapdown roll stabilized platform. The three-axis acceleration measurement range of the MIMU installed in the passive semi-strapdown roll stabilized platform were ±10*g*, and the range of the three-axis gyroscope was ±400 °/s.

The passive semi-strapdown roll stabilized platform based on the bearing nested structure and single bearing structure was separately installed on the vehicle turntable for testing. In order to simulate the high-speed rotation of the projectile, the high-precision vehicle turntable was equipped to rotate at 15 r/s, and the pitch and yaw directions were the same as the vehicle system SPAN-LCI, with a test time of 40 s.

**Figure 18:**
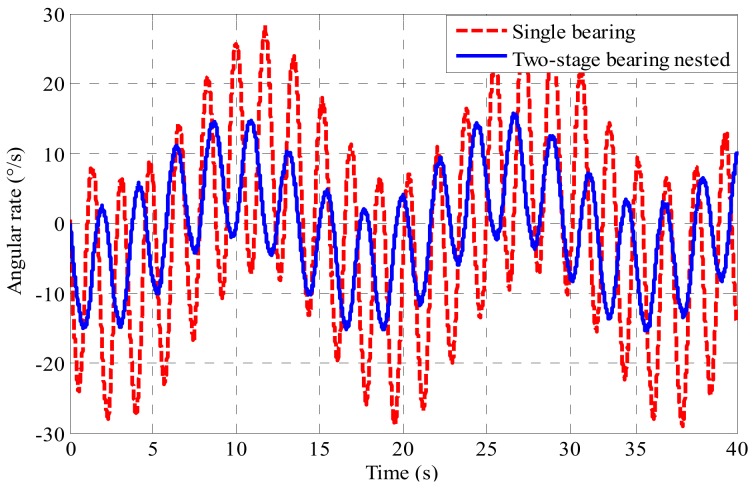
Actual value of the platform inner cylinder swing angular rate based on the single bearing and two-stage bearing nested structure under 900 r/min.

The inner cylinder roll rate of the passive semi-strapdown roll stabilized platform based on the bearing nested structure and the single bearing structure, respectively, is shown in Figure 18.

Due to the different bearing structures of the platform, the output waveforms of the two angular rates are different. By comparing the angular rates of Figure 18, it is found that the angular rate of the platform based on bearing nested structure decreases to 50% of that based on the single bearing structure. The proposed method of the bearing nested method can reduce the angular rate from around 35 °/s to around 15 °/s, and it also can reduce the swinging frequency of the inner cylinder. Because of the existence of the error angle of the system’s axial installation, it is impossible to ensure that the bearing only had radial pressure during the test, which would cause the bearing friction moment to change, and make the theoretical value and the experimental value different, leading to a slight difference between the actual value of the angular output rate and the theoretical value. The values calculated with the model agree well with the experimental results, which indicates that the proposed method is effective, and creates a condition for selecting sensors with a smaller range and higher accuracy.

**Figure 19:**
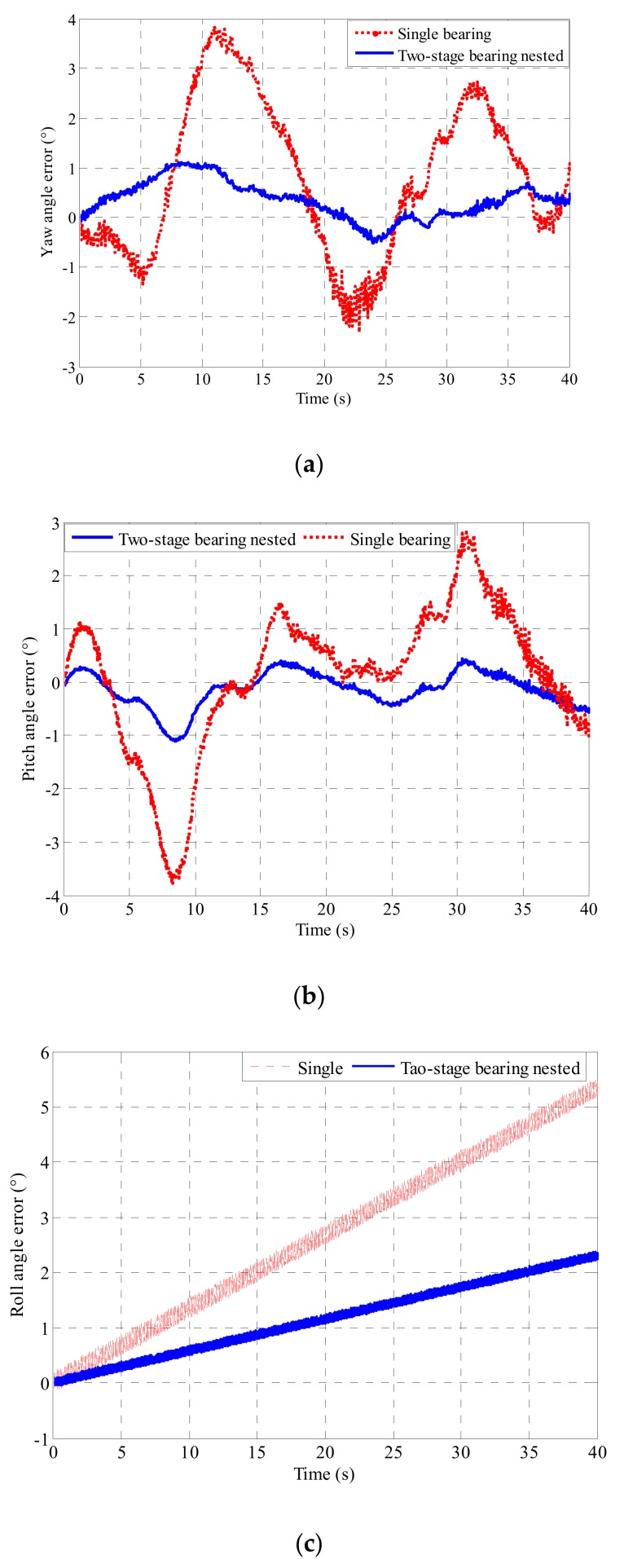
(**a**) The yaw angle error of the platform based on the single bearing and two-stage bearing nested structure. (**b**) The pitch angle error of the platform based on the single bearing and two-stage bearing nested structure. (**c**) The roll angle error of the platform based on the single bearing and two-stage bearing nested structure.

**Figure 20:**
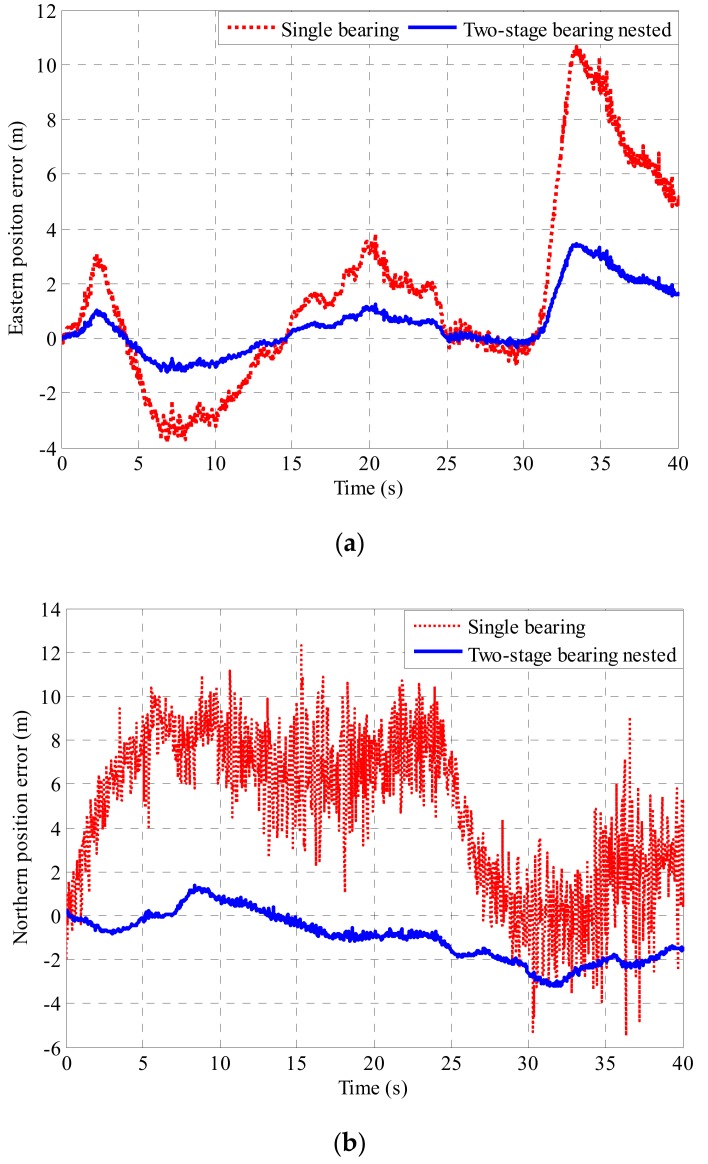

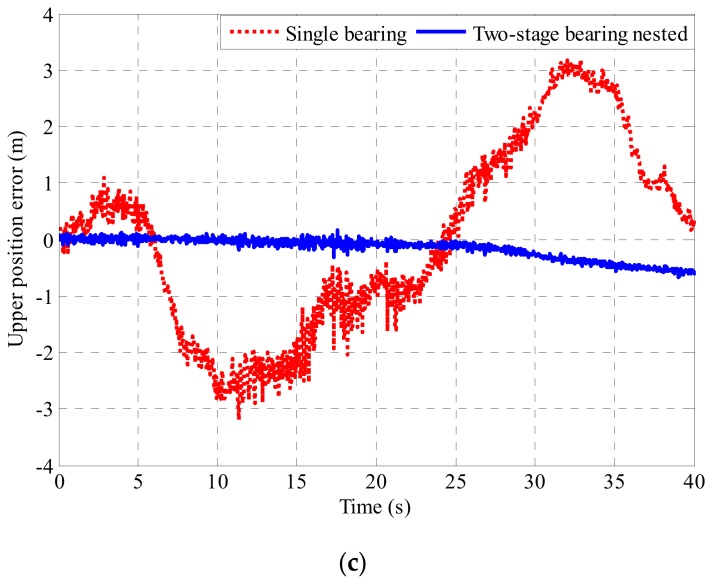
(**a**) The eastern position error of the platform based on the single bearing and two-stage bearing nested structure. (**b**) The northern position error of the platform based on the single bearing and two-stage bearing nested structure. (**c**) The upper position error of the platform based on the single bearing and two-stage bearing nested structure.

The data measured by the passive semi-strapdown roll stabilized platform based on the bearing nested structure and the single bearing are respectively combined with the relative rotation angle measured by the optical encoder to perform attitude and position calculation. The resulting three-dimensional attitude error and three-dimensional position error are shown in Figure 19 and Figure 20.

As can be seen from Figure 19 and Figure 20, in the actual environment of high-speed rotation, the introduction of the bearing nested structure in a high-speed rotating environment made the swing of the inner cylinder of the passive semi-strapdown roll stabilized platform more stable, and the measured results are in good agreement with the simulation results and theoretical calculation results. The passive semi-strapdown roll stabilized platform based on the bearing nested structure can reduce the yaw angle error from 3.5° to 1°, the pitch angle error from 3.7° to 1.1°, and the roll angle error from 5.3° to 2.3°. The passive semi-strapdown roll stabilized platform based on the bearing nested structure can reduce the eastern position error from 11 to 3 m, the northern position error from 11 to 3 m, and upper position error from 3 to 0.5 m. This shows that the measurement accuracy of the platform based on the bearing nested structure is higher, the position and attitude errors measured of the platform based on the bearing nested structure decreased to more than 50% of that based on the single bearing structure. The application of the bearing nested structure could improve the measurement accuracy of the passive semi-strapdown roll stabilized platform.

## 7. Conclusions

Based on the introduction of a passive semi-strapdown roll stabilized platform with a unique structure, this paper focuses on the effect of the rolling isolation device on the stability of the platform. In order to make the semi-strapdown roll stabilized platform more stable and further reduce the swing angular rate of the platform, the bearing structure of the semi-strapdown roll stabilized platform was optimized and improved. This paper proposed a bearing nested structure that could reduce the frictional moment of the stabilized platform. Using the existing friction moment calculation methods for reference, the dynamic modeling of the friction moment of the double-stage and multi-stage bearing nested structures was carried out. The theoretical value of the friction moment of the bearing nested structure in a missile-borne environment was obtained, and a series of simulation analysis and ground semi-physical test verification were carried out. The simulation and test results show that the proposed bearing nested structure had a better effect of isolating the projectile rolling angular rate.

This paper introduced a passive semi-strapdown roll stabilized platform with unique structure which could be applied to the measurement of a high-rolling projectile. The force analysis of the projectile and passive semi-strapdown roll stabilized platforms inner cylinder was carried out, and the dynamic equation of the platform inner cylinder under the action of air lift was established. On this basis, the influence of the bearing device on the stability of the platform was studied; the traditional single bearing was improved; a bearing nested structure was proposed; and the theoretical model of the friction moment of the bearing nested structure was established. Through a series of simulation analysis and experimental verification, the following conclusions can be drawn:

Compared with the traditional single bearing connection mode, the bearing nested structure has the following advantages:

1. Compared with the method of using only a single bearing, the scheme proposed in this paper has complementary advantages and can be better applied to the actual application environment Combining the large axial bearing capacity of angular contact ball bearings with the characteristic of the low cost and low friction coefficient of deep groove ball bearings to achieve the complementary advantages of bearing nested structure.
2. The friction moment of the passive semi-strapdown roll stabilized platform is reduced effectively. The bearing nested structure effectively solves the problem of large friction of a single bearing in high speed and high overload environments by adopting the way of “multi-stage isolation and indirect drive”, and significantly reduces the swing angular rate of the platform inner cylinder, which is beneficial to realize the high-precision navigation solution of the platform.
3. Improves the anti-overload capability and environmental adaptability of the platform. The bearing nested structure improves the axial overload resistance of the whole platform by means of multiple bearings bearing together.

## Figures and Tables

**Table 1:** Calculation method of friction variable.

Bearing Type	Rolling Friction Variable $G_{\mathrm{rr}}$	Sliding Friction Variable $G_{\mathrm{sl}}$
Deep groove ball bearing	$G_{\mathrm{rr}}=R_{1}d_{m}{\left(F_{r}+\frac{R_{2}}{\sin\alpha_{F}}\right)}^{0.54}$ $\alpha_{F}=24.6\times {\left(F_{a}/C_{0}\right)}^{0.24}$	$G_{\mathrm{sl}}=R_{1}d_{m}^{-0.145}{\left(F_{r}^{5}+\frac{S_{2}d_{m}^{1.5}}{\sin\alpha_{F}}F_{a}^{4}\right)}^{1/3}$
Angular contact ball bearing	$G_{\mathrm{rr}}=R_{1}d_{m}^{1.97}{\left[F_{r}+F_{g}+R_{2}F_{a}\right]}^{0.54}$ $F_{g}=R_{3}d_{m}^{4}n^{2}$	$G_{\mathrm{sl}}=S_{1}d_{m}^{0.26}\left[{\left(F_{r}+F_{g}\right)}^{4/3}+S_{2}F_{a}^{4/3}\right]$ $F_{g}=S_{3}d_{m}^{4}n^{2}$

**Table 2:** Bearing basic parameters.

Parameter	Deep Groove Ball Bearing SKF6200	Angular Contact Ball Bearing SKF7008AC
Outer diameter *D* (mm)	30	68
Inner diameter *d* (mm)	10	40
Limit speed (r·min^−1^)	34,000	26,000
Rated static load C_0_ (kN)	2.36	5.3
Friction coefficient μ	0.0015	0.0020
*R*_1_	3.9 × 10^−7^	5.03 × 10^−7^
*R*_2_	1.7	1.97
*R*_3_	/	1.90 × 10^−12^
*S*_1_	3.23 × 10^−3^	1.30 × 10^−2^
*S*_2_	36.5	0.68
*S*_3_	/	1.91 × 10^−12^

**Table 3:** Material parameters and properties of the bearing structure.

Material	Elastic Modulus/GPa	Density/(kg·m^−3^)	Poisson’s Ratio	Yield Strength/MPa
GCr15 bearing steel	210	7810	0.29	1458

**Table 4:** Relevant parameters of platform inner cylinder.

Parameter	Numerical Value
m/kg	1
$J_{o}$/(kg·mm^2^)	5860
L/mm	14
*g*/(m/s^2^)	9.8

## References

[1] Morrison P.H., Amberntson D.S. (2015). Guidance and control of a cannon-launched guided projectile. J. Spacecr. Rocket..

[2] Raul C., Luis C. (2018). Hybridized attitude determination techniques to improve ballistic projectile navigation, guidance and control. Aerosp. Sci. Technol..

[3] Frank F. (2011). Guidance and control of a projectile with reduced sensor and actuator requirements. J. Guid. Control Dyn..

[4] Du J., Guo Y., Lin Y. A real-time temperature compensation algorithm for a force-rebalanced MEMS capacitive accelerometer based on resonant frequency. Proceedings of the 2017 IEEE 12th International Conference on Nano/Micro Engineered and Molecular Systems (NEMS).

[5] Wang W., He S. (2009). Development of MEMS inertial instrument technology. Missiles Space Veh..

[6] Guo D. (2019). Weapon-target assignment for multi-to-multi interception with grouping constraint. IEEE Access.

[7] Titterton D.H., Weston J.L. (2004). Strapdown Inertial Navigation Technology.

[8] Xiong H.L., Mai Z.Z., Tang J., He F. (2019). Robust GPS/INS/DVL navigation and positioning method using adaptive federated strong tracking filter based on weighted least square principle. IEEE Access.

[9] Frank F., James D., Ilmars C. (2015). Flight performance of a small diameter munition with a rotating wing actuator. J. Spacecr. Rocket..

[10] Yang B., Wang Y., Xue L., Shan B., Wang B. (2018). Accurate integrated position and orientation method for vehicles based on strapdown inertial navigation system/Doppler radar. Meas. Control.

[11] Fu Q., Liu Y., Liu Z., Li S., Guan B. (2018). Autonomous in-motion alignment for land vehicle strapdown inertial navigation system without the aid of external sensors. J. Navig..

[12] Feng W.U., Qin Y.Y., Cheng Y. (2013). Transfer alignment for missile-borne SINS using airborne GPS on moving base. J. Chin. Inert. Technol..

[13] Luisa F.D., Frank E. (2016). Position estimation for projectiles using low-cost sensors and flight ynamics. J. Aerosp. Eng..

[14] Qian Z., Lei W., Zengjun L., Peide F. (2015). An accurate calibration method based on velocity in a rotational inertial navigation system. Sensors.

[15] Zhang Q., Wang L., Liu Z., Zhang Y. (2016). Innovative self-calibration method for accelerometer scale factor of the missile-borne rins with fiber optic gyro. Opt. Express.

[16] Boronakhin A.M., Podgornaya L.N., Bokhman E.D., Filipenya N.S., Filatov Y.V., Shalymov R.B., Larionov D.Y. (2011). Mems-based inertial system for railway track diagnostics. Gyroscopy Navig..

[17] Li S.T., Gao Y.B., Liu M. (2019). Multistage attitude determination alignment for velocity-aided in-motion strapdown inertial navigation system with different velocity models. Sensors.

[18] Wang Y.F., Sun F.C. (2012). Central difference particle filter applied to transfer alignment for SINS on missiles. IEEE Trans. Aerosp. Electron. Syst..

[19] Xu M.M., Bu X.Z., Yu J., He Z.L. (2018). Spinning projectile’s attitude measurement with LW infrared radiation under sea-sky background. Infrared Phys. Technol..

[20] Zhao H., Su Z., Liu F.C., Li C. (2019). Magnetometer-based phase shifting ratio method for high spinning projectile’s attitude measurement. IEEE Access.

[21] Long D.F., Zhang X.M., Wei X.H., Luo Z.L., Cao J.Z. (2018). A fast calibration and compensation method for magnetometers in strap-down spinning projectiles. Sensors.

[22] Duan X.M., Liu J., Li J., Yang W. (2014). Design and test of platform with partial strapdown inertial navigation system for guided projectile. Missiles Space Veh..

[23] Duan X.M., Liu J., Li J. (2014). IInfluence of air lift on the stability of passive partial strapdown platform. Acta Armamentarii.

[24] Duan X.M., Li J., Liu J. (2014). Research on the dynamic model of a partial strapdown platform and the impact analysis of pitching angle and the stability of platform. Acta Armamentarii.

[25] Florian S., Theodoulis S., Wernert P., Zasadzinski M., Boutayeb M. (2017). Flight dynamics modeling of dual-spin guided projectiles. IEEE Trans. Aerosp. Electron. Syst..

[26] Yang H.S., Deng S.E., Xia X.T., Zheng X.P., Liang B. (2005). Development of simulation technology of rolling bearing in SKF. Bearing.

[27] Das K., Batra R.C. (2009). Symmetry breaking, snap-through and pull-in instabilities under dynamic loading of microelectromechanical shallow arches. Smart Mater. Struct..

[28] Firouzian-Neja A.C., Bowen S. (2019). Bi-stable hybrid composite laminates containing metallic strips: An experimental and numerical investigation. Smart Mater. Struct..

[29] Kanatsu M., Ohta H. (2008). Running torque of ball bearings with polymer lubricant (running torque formulas of deep groove ball bearings under axial loads). J. Tribol..

[30] Cheng F.Z., Qu L.Y., Qiao W., Hao W. (2019). Enhanced particle filtering for bearing remaining useful life prediction of wind turbine drivetrain gearboxes. IEEE Trans. Ind. Electron..

[31] Wonil K., Lee J., Lee Y.B. (2019). Theoretical and experimental approach to ball bearing frictional characteristics compared with cryogenic friction model and dry friction model. Mech. Syst. Signal Process..

[32] Florian S., Florian G., Martin O., Stuart T. (2019). Challenges of friction reduction of engine plain bearings—Tackling the problem with novel bearingmaterials. Tribol. Int..

[33] Harris T.A. (1984). Rolling Bearing Analysis.

[34] Qin W.Y., Qin H., Zheng H.B., Zhang Z.Y. (2019). The coupled effect of bearing misalignment and friction on vibration characteristics of a propulsion shafting system. Proc. Inst. Mech. Eng. Part M.

[35] Qu J., Wang W., Han H.T., Liu X.W. (2016). Study on contact analysis of high angular contact ball bearings based on ANSYS. Mach. Des. Manuf..

